# The Effect of Prolonged Duration of Intensity Modulated Radiotherapy for Nasopharyngeal Carcinoma

**DOI:** 10.3389/fonc.2021.648637

**Published:** 2021-09-14

**Authors:** Yi-Jun Hua, Yan-Feng Ou-Yang, Xiong Zou, Le Xia, Dong-Hua Luo, Ming-Yuan Chen

**Affiliations:** ^1^Department of Nasopharyngeal Carcinoma, Sun Yat-sen University Cancer Center, Guangzhou, China; ^2^Sun Yat-sen University Cancer Center, State Key Laboratory of Oncology in South China, Collaborative Innovation Center for Cancer Medicine, Guangzhou, China; ^3^Guangdong Key Laboratory of Nasopharyngeal Carcinoma Diagnosis and Therapy, Guangzhou, China

**Keywords:** nasopharyngeal carcinoma, intensity-modulated radiotherapy, prognosis, radiation, chemotherapy, duration

## Abstract

**Purpose:**

Radiotherapy is the most important primary treatment for patients with nasopharyngeal carcinoma. Generally, the treatment duration of radiotherapy takes six or six and half weeks with 30 to 33 fractions. The current study was conducted to evaluate the association between prognosis and the duration of radiotherapy in nasopharyngeal carcinoma patients.

**Methods:**

Patients with primary nasopharyngeal carcinoma who were treated with intensity-modulated radiotherapy and concurrent cisplatin-based chemotherapy, with or without induction chemotherapy between January, 2008 and December, 2013 at a single institution were retrospectively reviewed.

**Results:**

In total, 1292 patients were included. At a median follow-up of 71.0 months (range 2.0–126.0 months), locoregional recurrence, distant failure and death were observed in 8.8%, 12.2% and 15.6% of all patients, respectively. Estimated 5-year locoregional relapse–free survival, distant metastasis–free survival, progression-free survival and overall survival in patients with radiation ≤ 7 weeks versus patients with radiation >7 weeks were: 93.2% versus 87.0% (P < 0.001), 89.4% versus 84.4% (P = 0.016), 79.8% versus 70.6% (P < 0.001) and 87.2% versus 78.4% (P < 0.001), respectively.

**Conclusions:**

Prolonged duration of radiotherapy with a significantly higher risk of distant metastasis and death in nasopharyngeal carcinoma patients. Understanding this point, healthcare providers should make efforts to avoid prolonged duration of radiotherapy to minimize the risk of treatment failure.

## Background

Nasopharyngeal carcinoma (NPC) is endemic in Southeast Asia, including Southern China. The current management of locoregionally advanced NPC is radiotherapy combined with cisplatin-based concurrent chemoradiotherapy. Radiotherapy still plays the most important role in the treatment of NPC. Intensity-modulated radiotherapy (IMRT), which can deliver high doses to the target while sparing adjacent tissues and organs, is now the mainstream radiation technique (1). With the development of imaging and modern radiation therapy techniques, the treatment outcomes have greatly improved in recent decades (2). Although NPC is highly sensitive to radiotherapy, a high failure rate is still noted in patients with advanced disease. There are some reasons for the high rate of failure. Advanced locoregional status is one of the most important factors (2, 3), and there are other factors, such as waiting time and radiation time, that could be managed (4).

Some studies have demonstrated that the treatment delay of radiotherapy was significantly associated with poorer survival rates in early stage head and neck cancer patients. Chen et al. also reported that a prolonged interval > 30 days between induction chemotherapy and radiotherapy was associated with a significantly higher risk of distant metastasis and death in NPC patients (5, 6). There are also some studies about the duration expansion of radiotherapy caused by different reasons. Studies have focused on the duration of radiotherapy and found that it is also an important factor that could affect treatment outcomes (4, 7). Therefore, it is important for health staff to be aware of the effects of radiation duration in clinical practice (6).

Induction chemotherapy has been demonstrated to effectively decrease the distant metastasis rate and improve survival (8, 9). Concurrent chemoradiotherapy (CCRT) has also indicated successfully decrease locoregional control, and now induction chemotherapy plus CCRT is recommended by National Comprehensive Cancer Network (NCCN) guidelines and has been practiced by clinical doctors (10). Although chemotherapy could bring benefits to patients, it also has side effects that could be a negative factor for the treatment outcome. Some reports also mentioned that the addition of concurrent chemotherapy also caused an increase in side effects, which is a common cause of the interruption of radiation, leading to a prolonged duration of radiation (4, 11).

The outbreak of COVID-19 is also associated with delay in treatment and loss of chances in terms of cancer treatment. Due to COVID-19, many cancer patients were affected for postponement of chemotherapy, radiotherapy and/or surgery, limited access to supportive care.

We hypothesized that a longer duration of radiotherapy (exceeding the normal span) would be correlated with worse survival in NPC patients treated with IMRT. Therefore, we conducted this retrospective study to assess the prognostic effect of the prolonged duration of radiotherapy in patients with NPC.

## Methods

### Ethical Consideration

The Clinical Research Ethics Committee of Sun Yat-sen University Cancer Center (SYSUCC) approved this retrospective review.

### Patients

We retrospectively reviewed the inpatient medical records of 1292 newly pathologically confirmed primary nasopharyngeal carcinoma patients without distant metastasis treated with IMRT at SYSUCC between January 2008 and December 2013. Patients who received cisplatin-based concurrent chemotherapy were included, while those who did not receive concurrent chemotherapy or those who received concurrent chemotherapy that was not cisplatin-based were excluded.

### Pretreatment Evaluation

All patients underwent complete physical examination, endoscopy, magnetic resonance imaging (MRI) of the head and neck, chest radiography, abdominal ultrasound, whole-body bone scanning, single photon emission computed tomography (SPECT) and dental assessment. Positron emission tomography and computed tomography.

(PET/CT) was performed when necessary. All the included patients were restaged according to the seventh edition of the American Joint Committee on Cancer (AJCC) staging system.

### Radiation

All patients received IMRT. The primary nasopharyngeal gross tumor volume (GTVnx) and the involved cervical lymph nodes were determined based on the MRI/CT and/or PET/CT imaging, clinical, and endoscopic findings. Enlarged retropharyngeal nodes together with primary gross tumor volume (GTV) were outlined as the GTVnx on the IMRT plans. The clinical tumor volume (CTV) represents the primary tumor with potential subclinical disease. The first clinical tumor volume (CTV1) was defined as the GTV plus a 0.5-1.0 cm margin (0.2 to 0.3 margin posteriorly) to encompass the high-risk sites of microscopic extension and the whole nasopharynx. Clinical target volume 2 (CTV2) was defined as the CTV1 plus a 0.5-1.0 cm margin (0.2 to 0.3 margin posteriorly) to encompass the low-risk sites of microscopic extension, the level of the lymph node, and the elective neck area (bilateral levels IIa, IIb, III, and Va were routinely covered for all N0 patients, whereas ipsilateral levels IV and Vb or supraclavicular fossae were also included for N1-3 patients). The prescribed dose was 66–70 Gy to the planning target volume (PTV), 60 Gy to PTV1, 54 Gy to PTV2, and 60–66 Gy to PTV of the involved cervical lymph nodes in 28 to 33 fractions. All patients were treated once daily, with five fractions weekly. Dose constraints to the critical structures were within the tolerance according to the RTOG 0225 protocol, and efforts were made to meet the criteria as closely as possible.

### Chemotherapy

During the study period, concurrent chemoradiotherapy (CCRT) ± induction chemotherapy (IC) for stage II to IV disease was recommended according to our institutional guidelines. One of the following three regimens of IC were used: PF (80-100 mg/m2 cisplatin on day 1 and 800 mg/m2/d fluorouracil civ on days 1–5), TP (75 mg/m2 docetaxel on day 1 and 75 mg/m2 cisplatin on day 1) and TPF (75 mg/m2 docetaxel on day 1, 75 mg/m2 cisplatin on day 1 and 800 mg/m2/d fluorouracil civ on days 1– 5); all regimens were repeated every 3 weeks for 2–3 cycles. The study-defined concurrent chemoradiotherapy regimen was 80–100 mg/m2 cisplatin on day 1 every 3 weeks for 2–3 cycles or 30 mg/m2 cisplatin weekly. Patients receiving other chemotherapy regimens or who received only one cycle of induction or concurrent chemotherapy were excluded from this study. Adjuvant chemotherapy was less often chosen because of poor compliance. Reasons for deviating from the institutional guidelines included organ dysfunction suggesting intolerance to chemotherapy, patient refusal, and the discretion of the doctors in individual cases.

### Anti-EGFR Therapy Delivery

Both nimotuzumab and cetuximab were not recommended for NPC patients by the guidelines at that time. Therefore, the use of anti-EGFR therapy was determined by the patients’ willingness and the experience of doctors. Intravenous nimotuzumab was administered at an initial dose of 200 mg weekly during the whole radiation period. Intravenous cetuximab was administered at an initial dose of 400 mg/m2 followed by 250 mg/m2 weekly throughout RT.

### Duration of Radiotherapy

The duration of radiotherapy was calculated from the start of radiotherapy to the end of radiotherapy. All patients received radiotherapy in 28 to 33 fractions. We used a cut-off point of more than 7 weeks to define a longer duration of radiotherapy.

### Follow-Up

Patient follow-up was measured from the first day of therapy to the day of the last examination or death. The patients were examined at least every 3 months during the first 2 years, with follow-up examinations every 6 months for 3 years or until death. The last follow-up date was 20 April 2019. Overall survival (OS) was calculated from day 1 after the completion of treatment to the last examination or death. Distant metastasis–free survival (DMFS) and locoregional relapse–free survival (LRRFS) were calculated from day 1 after the completion of treatment to first distant metastasis and locoregional relapse, respectively; progression–free survival (PFS) was calculated from day 1 after the completion of treatment to locoregional relapse, distant relapse or tumor-related death, whichever occurred first.

### Statistical Analysis

The clinicopathological characteristics of the participants were assessed, and the differences in these characteristics were compared by χ2 test for categorical variables and t-test for continuous variables. Logistic regression analysis was used to identify confounders between the treatment groups. LRRFS, DMFS, PFS and OS were calculated using the Kaplan-Meier method. The differences in LRRFS, DMFS, PFS and OS between the two groups were tested using the log-rank test. Multivariate analysis was performed using the Cox proportional hazards model. All statistical analyses were performed using SPSS 21.0 statistical software (Chicago, IL, USA). *P* < 0.05 was considered statistically significant.

## Results

### Patient Characteristics

A total of 1292 NPC patients who were treated with IMRT between January 2008 and December 2013 at SYSUCC were analyzed in this study.

Among the 1292 patients, 290 were female and 1002 were male. The mean age at the time of reirradiation was 43.5 years (SD=10.2) for radiation duration ≤ 7 weeks and 45.8 years (SD=10.6) for radiation duration >7 weeks. All 1292 patients received cisplatin-based concurrent chemotherapy, and 647 patients received two or three courses of induction chemotherapy. 883 patients received radiotherapy within a duration less than 7 weeks, and 409 patients within a duration more than 7 weeks. Of the 409 patients, 253 (61.9%) patients experienced a relative long duration because of long-term public holidays (May Day holidays, National Days and Spring Festival holiday). The characteristics of the patients are shown in **Table 1**.

**Table 1:** Characteristics of the 1292 patients.

Characteristics	Duration of radiotherapy ≤ 7 weeks(883 patients)	Duration of radiotherapy > 7 weeks(409 patients)	P-value
Age (years)	43.5±10.2	45.8±10.0	0.684
Sex			0.753
Female	196 (22.2%)	94 (23.0%)	
Male	687 (77.8%)	315 (77.0%)	
T category			0.023
T1	56 (6.3%)	16 (3.9%)	
T2	122 (13.8%)	69 (16.9%)	
T3	478 (54.1%)	197 (48.2%)	
T4	227 (25.7%)	127 (31.1%)	
T category			0.796
T1+2	178 (20.1%)	85 (20.8%)	
T3+4	705 (79.9%)	324 (79.2%)	
N category			0.065
N0	100 (11.3%)	29 (7.1%)	
N1	375 (42.5%)	167 (40.8%)	
N2	332 (37.6%)	174 (42.5%)	
N3	76 (8.6%)	39 (9.5%)	
Clinical Stage			0.055
II	76 (8.6%)	36 (8.8%)	
III	526 (59.6%)	216 (52.8%)	
IV	281 (31.8%)	157 (38.4%)	
Chemotherapy			
CCRT	454 (51.4%)	191 (46.7%)	0.115
IC+CCRT	429 (48.6%)	218 (53.3%)	
Anti-EGFR			<0.001
Without	595 (67.4%)	355 (86.8%)	
With	288 (32.6%)	54 (13.2%)	

IC, induction chemotherapy; CCRT, concurrent chemoradiotherapy.

At a median follow-up of 71.0 months (range 2.0–126.0 months), the 1-, 3-, and 5-year follow-up rates were 99.2%, 97.8% and 91.2%, respectively. At the time of the analysis, 114 (8.8%) patients had locoregional failure, 157 (12.2%) developed distant metastases, and 202 (15.6%) died.

### Patient Characteristics and Association With the Duration of Radiotherapy

The patient characteristics for the entire included cohort are displayed in **Table 1**. Patients with a duration of more than 7 weeks tended to receive anti-EGFR (P < 0.001). We also found that patients with a duration of more than 7 weeks were more likely to have advanced T stages (P = 0.023). However, after we recategorized T stage as a binary variable (T1–2 and T3–4) before entering the Cox regression, the correlation between T stage and the interval became statistically nonsignificant (Pearson chi-square test, P = 0.796). For the other remaining characteristics, there were no significant correlations between them and the radiation duration.

### Prognosis

**Figure 1:**
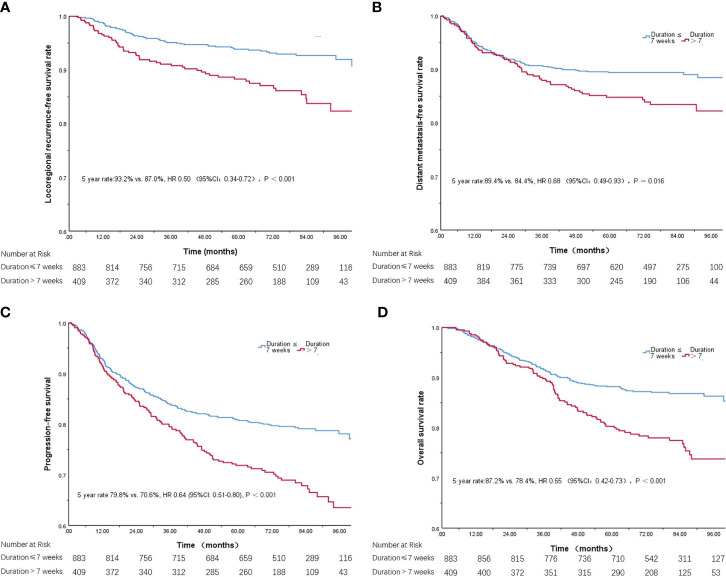
Kaplan-Meier curves of locoregional recurrence-free survival **(A)**, distant metastasis-free survival **(B)**, progression-free survival **(C)**, and overall survival **(D)**, with radiation duration ≤ 7 weeks or >7 weeks.

The 5-year overall survival rate was significantly lower for patients with radiation duration >7 weeks than for those completing radiation within 7 weeks (87.2% *vs.* 78.4%, P < 0.001; **Figure 1D**). The 5-year locoregional recurrence-free rate (93.2% *vs.* 87.0%, P < 0.001; **Figure 1A**), 5-year distant metastasis-free survival rate (89.4% *vs.* 84.4%, P = 0.026; **Figure 1B**) and progression-free survival rate (79.8% *vs.* 70.6%, P < 0.001; **Figure 1C**) were also significantly lower for patients with radiation duration ≤ 7 weeks than for those with duration > 7 weeks.

All 1292 patients were analyzed by univariate and multivariable Cox regression models. We included radiation duration (≤ 7 weeks vs. > 7 weeks), sex, age, T stage, N stage, anti-EGFR treatment (received vs. did not receive) and concurrent chemotherapy (with or without induction chemotherapy) in the model. Univariate Cox regression analysis showed that radiation duration (≤ 7 weeks vs. > 7 weeks), sex, age, T stage, N stage, and anti-EGFR treatment were found to have prognostic significance for OS. For DMFS, radiation duration (≤ 7 weeks vs. > 7 weeks), sex, N stage and received anti-EGFR treatment were found to have prognostic significance. Radiation duration (≤ 7 weeks vs. > 7 weeks), age, N stage and received anti-EGFR treatment were found to have prognostic significance for PFS. Only radiation duration (≤ 7 weeks vs. > 7 weeks) and N stage had prognostic significance for LRRFS (**Table 2**). Multivariate analysis indicated that radiation duration (≤ 7 weeks vs. > 7 weeks) was an independent prognostic factor for DMFS, LRRFS, PFS and OS (**Table 3**).

**Table 2:** Prognostic factors associated with overall survival by univariate Cox regression model (N=1292).

	DMFS				LRRFS				PFS				OS			
	B	SE	HR (95%CI)	*P*	B	SE	HR (95%CI)	*P*	B	SE	HR (95%CI)	*P*	B	SE	HR (95%CI)	*P*
Sex																
Female		1				1				1				1		
Male	-0.802	0.245	0.448 (0.278-0.725)	*0.001*	0.185	0.211	1.203 (0.795-1.819)	0.381	-0.325	0.147	0.722 (0.542-0.963)	0.027	-0.457	0.190	0.633 (0.436-0.919)	0.016
Radiation duration																
≤ 7 weeks		1				1				1				1		
> 7 weeks	-0.392	0.163	0.676 (0.491-0.931)	0.016	-0.704	0.188	0.495 (0.342-0.715)	<0.001	-0.452	0.115	0.636 (0.507-0.798)	<0.001	-0.591	0.142	0.554 (0.419-0.731)	<0.001
Age (continuous)	0.004	0.008	1.004 (0.989-1.020)	0.583	0.009	0.009	1.009 (0.990-1.027)	0.352	0.015	0.006	1.015 (1.004-1.026)	0.007	0.027	0.007	1.027 (1.013-1.041)	<0.001
Tumor stage																
T1		1				1				1				1		
T2	-0.295	0.380	0.745 (0.353-1.570)	0.439	-0.323	0.442	0.724 (0.305-1.721)	0.465	0.050	0.396	1.051 (0.484-2.285)	0.900	-0.812	0.349	0.444 (0.224-0.881)	0.020
T3	-0.381	0.264	0.684 (0.404-1.146)	0.148	0.133	0.265	1.142 (0.679-1.921)	0.616	-0.126	0.277	0.882 (0.513-1.517)	0.650	-0.769	0.226	0.463 (0.298-0.721)	0.001
T4	-0.299	0.181	0.742(0.520-1.056)	0.098	-0.456	0.221	0.634 (0.411-0.977)	0.039	-0.577	0.162	0.562 (0.409-0.772)	<0.001	-0.930	0.155	0.3695 (0.291-0.535)	<0.001
Node stage																
N0		1				1				1				1		
N1	-1.353	0.338	0.258 (0.133-0.501)	<0.001	-1.477	0.467	0.228 (0.090-0.580)	0.002	-1.238	0.262	0.290 (0.174-0.485)	<0.001	*-1.531*	0.331	0.216 (0.113-0.414)	<0.001
N2	-1.723	0.243	0.179 (0.111-0.288)	<0.001	-0.899	0.288	0.407 (0.231-0.716)	0.002	-1.038	0.176	0.339 (0.240-0.478)	<0.001	*-1.309*	0.206	0.270 (0.180-0.405)	<0.001
N3	-0.813	0.209	0.444 (0.295-0.668)	<0.001	-0.634	0.282	0.531 (0.305-0.923)	0.025	-0.604	0.167	0.547 (0.394-0.759)	<0.001	*-0.812*	0.194	0.444 (0.304-0.649)	<0.001
Anti-EGFR																
Without		1				1				1				1		
With	0.440	0.203	1.553 (1.043-2.313)	*0.030*	-0.135	0.207	0.874 (0.583-1.312)	0.516	0.353	0.140	1.424 (1.081-1.875)	0.012	0.664	0.193	1.943 (1.331-2.835)	0.001
Induction chemotherapy																
CCRT		1				1				1				1		
IC+CCRT	0.116	0.161	1.123 (0.819-1.538)	0.472	-0.368	0.190	0.692 (0.477-1.005)	0.053	-0.129	0.114	0.879 (0.703-1.098)	0.256	-0.226	0.141	0.798 (0.605-1.053)	0.110

OS, Overall survival; DMFS, Distant metastasis–free survival; LRRFS, locoregional relapse–free survival; PFS, progression–free survival; IC, induction chemotherapy; CCRT, Concurrent chemoradiotherapy.

**Table 3:** Multivariate analyses of factors based on the Cox regression model.

Outcomes	Variables in the final model	B	SE	HR (95%CI)	P
**DMFS**					
	Tumor stage				
	T1				
	T2	-0.310	0.387	0.734 (0.343-1.568)	0.424
	T3	-0.695	0.272	0.499 (0.293-0.850)	0.011
	T4	-0.366	0.185	0.694 (0.483-0.997)	0.048
	Node stage				
	N0				
	N1	-1.502	0.344	0.223 (0.113-0.437)	<0.001
	N2	-1.854	0.248	0.157 (0.096-0.255)	<0.001
	N3	-0.908	0.212	0.403 (0.266-0.612)	<0.001
	Induction chemotherapy				
	CCRT				
	IC+CCRT	0.405	0.168	1.500 (1.078-2.086)	0.016
	Radiation duration				
	≤ 7 weeks				
	> 7 weeks	-0.362	0.165	0.696 (0.504-0.962)	0.028
**LRRFS**					
	Node stage				
	N0				
	N1	-1.346	0.481	0.260 (0.101-0.669)	0.005
	N2	-0.823	0.292	0.439 (0.248-0.779)	0.005
	N3	-0.603	0.284	0.547 (0.314-0.955)	0.034
	Radiation duration				
	≤ 7 weeks				
	> 7 weeks	-0.637	0.190	0.529 (0.365-0.768)	<0.001
					
**PFS**					
	Age (continuous)	0.011	0.005	1.011 (1.001-1.022)	0.037
	Tumor stage				
	T1				
	T2	-0.622	0.297	0.537 (0.300-0.962)	0.036
	T3	-0.463	0.180	0.629 (0.442-0.895)	0.010
	T4	-0.565	0.131	0.568 (0.440-0.734)	<0.001
	Node stage				
	N0				
	N1	-1.299	0.266	0.273 (0.162-0.459)	<0.001
	N2	-1.127	0.179	0.324 (0.228-0.460)	<0.001
	N3	-0.634	0.169	0.530 (0.381-0.739)	<0.001
	Radiation duration				
	≤ 7 weeks				
	> 7 weeks	-0.382	0.117	0.682 (0.543-0.857)	<0.001
**OS**					
	Age (continuous)	0.021	0.007	1.021 (1.008-1.035)	0.007
	Tumor stage				
	T1				
	T2	-0.765	0.355	0.465 (0.232-0.932)	0.031
	T3	-0.942	0.233	0.390 (0.247-0.615)	<0.001
	T4	-0.943	0.159	0.393 (0.288-0.536)	<0.001
	Node stage				
	N0				
	N1	-1.668	0.336	0.189 (0.098-0.364)	<0.001
	N2	-1.404	0.210	0.246 (0.163-0.371)	<0.001
	N3	-0.863	0.196	0.422 (0.287-0.620)	<0.001
	Radiation duration				
	≤ 7 weeks				
	> 7 weeks	-0.505	0.143	0.604 (0.456-0.799)	<0.001

OS, Overall survival; DMFS, Distant metastasis–free survival; LRRFS, locoregional relapse–free survival; PFS, progression–free survival.

## Discussion

It was expected that a prolonged radiation duration was related to unfavorable clinical outcomes based on several studies that confirmed the benefit on tumor control and survival when radiotherapy for head and neck cancer was given without interruptions (4, 6, 7). Our study demonstrated that a longer duration of radiotherapy was an independent factor in NPC patients. Both univariate and multivariate analyses revealed that prolonged radiation duration > 7 weeks was a negative prognostic factor for DMFS, LRRFS, PFS and OS for NPC compared to the interval ≤ 7 weeks. It is important to raise awareness of prolonged radiation time for decision makers in clinical practice.

The general consensus for radiotherapy is that treatment should be given without interruptions. In real clinical practice, there are always some reasons for prolonged radiation time. Several studies have also demonstrated the impact of a longer duration of radiation treatment on local failure risk and overall survival in patients with NPC and other types of cancers (12–15), and in these studies, radiation is conventional radiotherapy. This phenomenon has been proven in both xenograft animal models and clinical studies with cervical cancer, bladder cancer and head and neck cancer (15–17). For NPC, a study published by Hong Kong researchers confirmed that interruptions in radiation and the prolongation of radiation adversely affect outcomes in radiotherapy (7). Other studies have also demonstrated the impact of prolonged radiation duration on local control risk and overall survival in patients with head and neck cancers (18, 19). We must note that previous studies were all based on conventional radiotherapy. Our present study is based on IMRT, and the results show that the estimated 5-year locoregional relapse-free survival, distant metastasis-free survival, progression-free survival and overall survival in patients with radiation ≤ 7 weeks *versus* patients with radiation > 7 weeks were 93.2% *versus* 87.0% (P < 0.001), 89.4% *versus* 84.4% (P = 0.016), 79.8% versus 70.6% (P < 0.001) and 87.2% *versus* 78.4% (P < 0.001), respectively. The results demonstrated that those patients who finished their radiotherapy on schedule had a better outcome than those who had interruptions during their radiotherapy due to any issues. The reason for the association between the prolonged duration and prognosis of NPC patients is complex. One possible explanation is as follows: when treatment is interrupted, the repopulation and recycling of tumor cells can occur (16), which is believed to be a significant risk for treatment failure; however, this explanation needs further study, especially since IMRT is currently popular in clinical practice and the basic radiation biology of IMRT is still not well clarified, which seems to make this issue slightly more complex.

A clear understanding of the factors associated with a prolonged waiting time can aid clinicians in providing better care (6). The causes of unplanned treatment interruptions are likely complex and multifactorial. In general, the acute toxicity caused by radiation and chemotherapy is responsible for unplanned treatment interruptions (20). Studies have demonstrated that concurrent chemotherapy increases acute toxicity over radiotherapy alone (4). The most common treatment-related side effects that lead to unplanned treatment interruptions are severe mucositis and skin reactions (4). Some comorbid conditions are associated with delayed wound healing, especially poor nutritional status, vascular disease, and diabetes mellitus (21). Cisplatin-based concurrent chemotherapy can cause nausea, vomiting and other complications, while nedaplatin can achieve similar treatment benefits without too many complications. Some research studies have already shown that concurrent chemotherapy is associated with the greatest duration of radiotherapy (4, 11). To minimize the negative effect, supportive medications to improve symptoms such as odynophagia and severe skin reactions should be provided as early as possible. Another reason may be caused by shortage of radiotherapy facilities, although they are available worldwide, but are often inadequate to the population demands placed on them due to an increasing number of patients since cancer incidence has increased in various parts of the world. However, we must note another special factor in China: national long-term holidays, such as May Day holidays, National Days and Spring Festival holiday. All these holidays will cause an interruption of radiation duration to more than 7 days. In the present study, 61.9% of NPC patients with prolonged duration of radiotherapy (radiation duration >7 weeks) were associated with these holidays since radiation ceased at that time.

The strength of this study was that it was based a large patient population and intensity-modulated radiotherapy. We must admit that our study was limited by its retrospective and single center nature without external validation of the results. First, the presented data were derived from a single institution located in an endemic area with expertise in NPC. Second, there was no randomization; therefore, some imbalance is inevitable. However, based on the results of the present study, launching a prospective randomized clinical trial to elucidate the relationship between radiation duration and prognosis in NPC patients may be ethically unacceptable.

## Conclusions

In conclusion, this study demonstrated that the prolonged duration of intensity-modulated radiotherapy is associated with a high risk of locoregional and distant failure and therefore the survival prognosis is worse for NPC patients, especially for patients with advanced N stages. Our present study may help clinical decision makers better understand this patient population and even apply these results to those with other head-and-neck cancers and take preventive measures to make optimal decisions on how to reduce the length of treatment in the future. And this also remind health workers should take proper solutions to minimize the disruptions during current pandemic of COVID-19.

## Data Availability Statement

The datasets presented in this study (RDDA2021001976) can be found in online repositories. The names of the repository/repositories and accession number(s) can be found below: http://www.researchdata.org.cn.

## Ethics Statement

The studies involving human participants were reviewed and approved by Sun Yat-sen University Cancer Center Research Ethics Committee. The patients/participants provided their written informed consent to participate in this study.

## Author Contributions

M-YC and Y-JH conceived the study. D-HL and XZ made substantial contributions to data acquisition, LX, D-HL and Y-FO-Y analyzed the data and performed interpretation of data. LX, D-HL and Y-JH were involved in drafting the manuscript. Y-JH edited the manuscript. All authors contributed to the article and approved the submitted version.

## Funding

This work was supported by Medical Scientific Research Foundation of Guangdong Province, China (NO.A2019395), the Program of Sun Yat-Sen University for Clinical Research 5010 program (No. 2019010).

## Conflict of Interest

The authors declare that the research was conducted in the absence of any commercial or financial relationships that could be construed as a potential conflict of interest.

## Publisher’s Note

All claims expressed in this article are solely those of the authors and do not necessarily represent those of their affiliated organizations, or those of the publisher, the editors and the reviewers. Any product that may be evaluated in this article, or claim that may be made by its manufacturer, is not guaranteed or endorsed by the publisher.
